# Hotspots of Malaria Transmission in the Peruvian Amazon: Rapid Assessment through a Parasitological and Serological Survey

**DOI:** 10.1371/journal.pone.0137458

**Published:** 2015-09-10

**Authors:** Angel Rosas-Aguirre, Niko Speybroeck, Alejandro Llanos-Cuentas, Anna Rosanas-Urgell, Gabriel Carrasco-Escobar, Hugo Rodriguez, Dionicia Gamboa, Juan Contreras-Mancilla, Freddy Alava, Irene S. Soares, Edmond Remarque, Umberto D´Alessandro, Annette Erhart

**Affiliations:** 1 Instituto de Medicina Tropical Alexander von Humboldt, Universidad Peruana Cayetano Heredia, Lima 31, Perú; 2 Research Institute of Health and Society (IRSS), Université catholique de Louvain, Brussels 1200, Belgium; 3 Department of Biomedical Sciences, Institute of Tropical Medicine, Antwerp 2000, Belgium; 4 Región de Salud Loreto, Iquitos, Loreto 160, Peru; 5 Departamento de Ciencias Celulares y Moleculares, Facultad de Ciencias y Filosofia, Universidad Peruana Cayetano Heredia, Lima 31, Peru; 6 Departamento de Análises Clínicas e Toxicológicas, Faculdade de Ciências Farmacêuticas, Universidade de São Paulo, São Paulo 05508–900, Brazil; 7 Department of Parasitology, Biomedical Primate Research Centre, 2280 GH Rijswijk, The Netherlands; 8 Disease Control and Elimination, Medical Research Council Unit, Fajara 220, The Gambia; 9 London School of Hygiene & Tropical Medicine, London WC1E 7HT, United Kingdom; Centro de Pesquisa Rene Rachou/Fundação Oswaldo Cruz (Fiocruz-Minas), BRAZIL

## Abstract

**Background:**

With low and markedly seasonal malaria transmission, increasingly sensitive tools for better stratifying the risk of infection and targeting control interventions are needed. A cross-sectional survey to characterize the current malaria transmission patterns, identify hotspots, and detect recent changes using parasitological and serological measures was conducted in three sites of the Peruvian Amazon.

**Material and Methods:**

After full census of the study population, 651 participants were interviewed, clinically examined and had a blood sample taken for the detection of malaria parasites (microscopy and PCR) and antibodies against *P*. *vivax* (PvMSP1_19_, PvAMA1) and *P*. *falciparum* (PfGLURP, PfAMA1) antigens by ELISA. Risk factors for malaria infection (positive PCR) and malaria exposure (seropositivity) were assessed by multivariate survey logistic regression models. Age-specific seroprevalence was analyzed using a reversible catalytic conversion model based on maximum likelihood for generating seroconversion rates (SCR, λ). SaTScan was used to detect spatial clusters of serology-positive individuals within each site.

**Results:**

The overall parasite prevalence by PCR was low, i.e. 3.9% for *P*. *vivax* and 6.7% for *P*. *falciparum*, while the seroprevalence was substantially higher, 33.6% for *P*. *vivax* and 22.0% for *P*. *falciparum*, with major differences between study sites. Age and location (site) were significantly associated with *P*. *vivax* exposure; while location, age and outdoor occupation were associated with *P*. *falciparum* exposure. *P*. *falciparum* seroprevalence curves showed a stable transmission throughout time, while for *P*. *vivax* transmission was better described by a model with two SCRs. The spatial analysis identified well-defined clusters of *P*. *falciparum* seropositive individuals in two sites, while it detected only a very small cluster of *P*. *vivax* exposure.

**Conclusion:**

The use of a single parasitological and serological malaria survey has proven to be an efficient and accurate method to characterize the species specific heterogeneity in malaria transmission at micro-geographical level as well as to identify recent changes in transmission.

## Introduction

Despite several decades of intense control efforts, malaria remains an important public health problem in Peru [1], mainly in the Department of Loreto in the Amazon region, which has historically accounted for most of the malaria burden within the country [2]. After malaria resurgence in the late 90s, with a peak of more than 120,000 slide confirmed cases in 1997 [1,3], the annual incidence in Loreto following intensified control activities decreased and stabilized at around 45,000–55,000 cases between 2002 and 2005 [2]. Between October 2005 and September 2010, increased support from international donors, e.g. the Global Fund-PAMAFRO Project [4], allowed the scale-up of comprehensive malaria control strategies in the Peruvian Amazon [5,6]. During this period, malaria declined drastically in Loreto from 54,291 reported clinical cases (25% due to *P*. *falciparum*) in 2005 to 11,604 cases (20% due to *P*. *falciparum*) in 2010, [2]. Since October 2010, malaria control activities are mainly supported by the Ministry of Health (MoH) budget, prioritizing passive and reactive case detection using standard light microscopy (LM) and treatment of confirmed infections. Unfortunately since 2012, malaria incidence in Loreto is rising again, with more than 25,000 cases reported [2].

Although Loreto is considered as a low endemic area [7], previous studies around Iquitos have reported high heterogeneity in malaria transmission at micro-geographical scale [8,9]. Even though this heterogeneity creates opportunities for targeted control interventions [10], the identification of hotspots of high malaria transmission intensity (MTI) is not straightforward. Choosing an appropriate method to measure MTI is key in assessing the risk of infection. Though the entomological inoculation rate (EIR) is usually considered as the gold standard for measuring MTI, in low transmission area this measure has proven to be insensitive and non cost-effective [11,12]. Incidence of confirmed clinical malaria has frequently been used for malaria risk stratification; however, its validity is affected by the differential acquisition of immunity inside and outside hotspots as well as contextual factors such as treatment seeking behaviors [10]. Although determining malaria parasite carriage (both symptomatic and asymptomatic infections) through large community surveys seem to be a more accurate method, this is still unable to capture seasonal variations [12,13]. Additionally, low-parasite-density infections would only be detected by highly sensitive diagnostic tests such as Polymerase Chain Reaction (PCR) [14].

Cross-sectional surveys using serological markers are increasingly proposed as useful alternatives for estimating MTI in low transmission settings, especially for the identification of malaria hotspots and related risk factors [15–19]. Age-stratified seroprevalence data have been used to model Sero-Conversion Rates (SCR) which are closely correlated with EIR, parasite prevalence rates [20–22] as well as related changes over time following specific interventions [22], seasonal or behavioral changes [23].

Following the recent changes in malaria control activities in the Peruvian Amazon region, we conducted a cross-sectional survey combining molecular and serological markers with the aim of characterizing the current malaria transmission patterns, detecting hotspots, and identifying recent changes in species-specific MTI in three sites around Iquitos city.

## Material and Methods

### Study area

**Fig 1:**
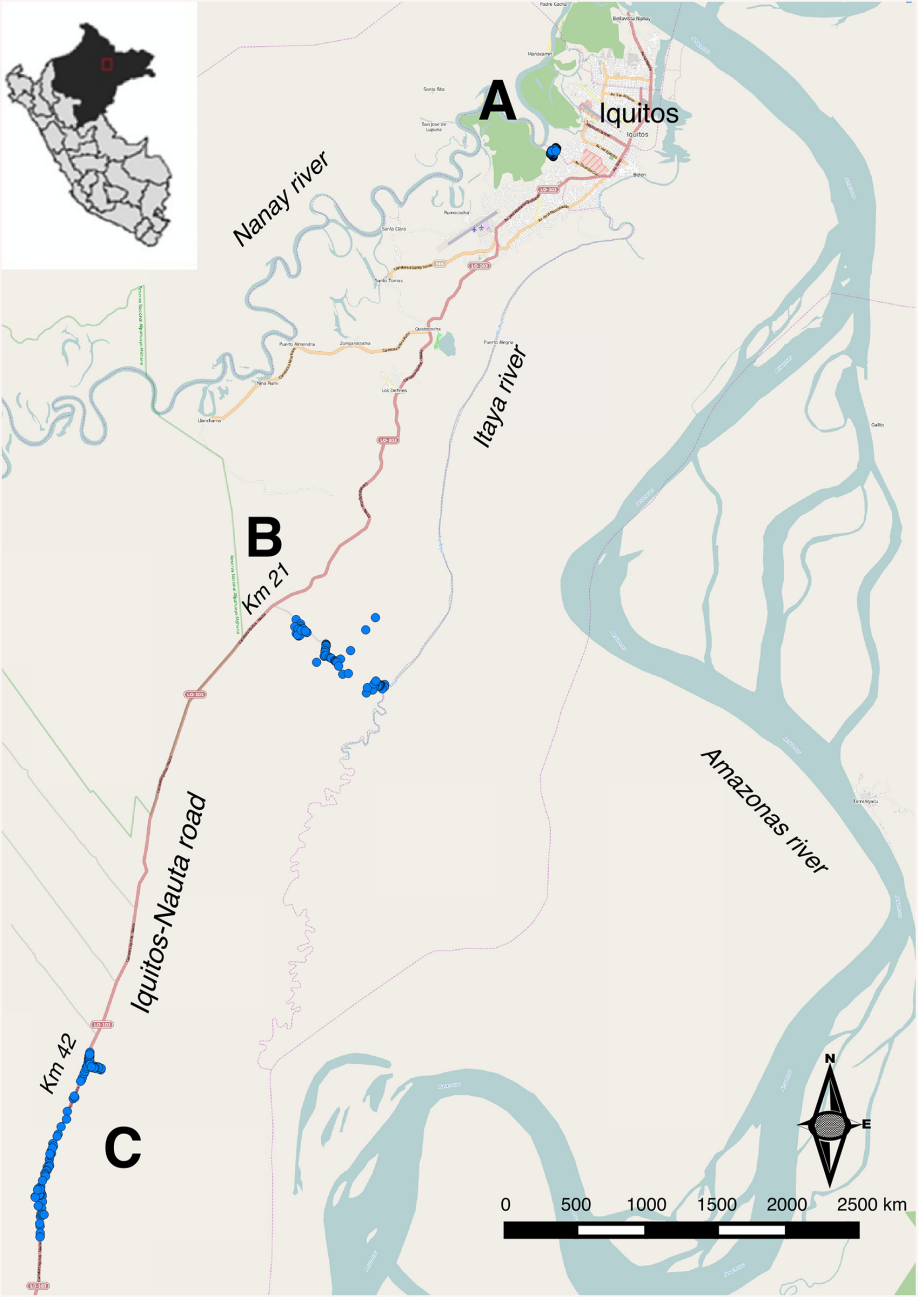
Study area in San Juan district, Loreto department, Northern Peruvian Amazon. *Eight communities in three sites were included.* Site A: Aguaje (AG), Agua Blanca (AB) and Pampachica (PA); Site B: El Milagro (EM), Villa Buen Pastor (BP) and San Carlos (SC); and Site C: San Lucas (SL), El Triunfo (ET).

The study was conducted in three different sites in San Juan (south of Iquitos city) (Fig 1), one of the most malaria endemic district in Loreto over the past five years. Study sites included communities which were established after the 1980s following the intensification of the deforestation and the extension of the road connecting Iquitos to Nauta district [24]. Site A included three contiguous peri-urban communities, i.e. Aguaje (AG), Agua Blanca (AB) and Pampachica (PA), located about 500 meters from the Nanay river, just on the administrative border between San Juan and Iquitos districts. The communities have well-constructed wooden houses (with 24 hour electric service) built on stilts as the river floods the area during the rainy season. Site B included three rural communities, i.e. El Milagro (EM), Villa Buen Pastor (BP) and San Carlos (SC), situated at 21 kilometers (km) from Iquitos on the Iquitos-Nauta district road, and then at 2, 4 and 9 km, respectively via a rural trail. The surrounding area is mainly composed by dense secondary growth vegetation with natural water bodies (swamps, ground pools, etc.) that enlarge during the rainy season. Among the three communities, only SC has direct access to the Itaya River. Site C was the farthest, consisting of two rural communities, i.e. San Lucas (SL) and El Triunfo (ET), with houses located on both sides of the Iquitos-Nauta road between the 42^nd^ and 48^th^ km. Several fish ponds are found along this section of the road. Unlike site A, communities in sites B and C have an important proportion of semi-closed wooden houses and no electricity. The study population was predominantly mestizos—referring to all Peruvians that cannot be clearly identified as belonging to any ethnic minority population- living mainly from small-scale agriculture, fishing, and small commercial activities.

The climate is tropical, warm and humid with a rainy season from November to May and a dry season from June to October. The annual average temperature is around 27°C, the relative humidity above 80%, and an average annual rainfall of 4 meters [25]. Malaria transmission in the area is perennial, with a peak between February and July [8]. *Anopheles darlingi* is the main malaria vector and is highly anthropophilic [26]. *P*. *vivax* and *P*. *falciparum* infections at district level occur at a ratio 5:1 and all age groups are at risk of infection, though adults more than children. Malaria surveillance relies on passive case detection (PCD) with microscopy. Patients presenting with fever or any other symptoms compatible with malaria are systematically tested by microscopy at health facilities and treated with chloroquine (CQ) for 3 days (10mg/g on days 1 and 2, and 5mg/kg on day 3), plus primaquine (PQ) for 7 days (0.5 mg/kg/day) if *P*. *vivax* malaria is confirmed, or with mefloquine (MQ) (12.5 mg/kg/day for 2 days) plus artesunate (AS) (4 mg/kg/day for 3 days) if *P*. *falciparum* malaria is confirmed. These treatment guidelines are in place since 2001, and all health facilities should perform directly observed therapy (DOT).

### Data collection

A complete census of the study population with collection of information on socio-demographic variables (age, gender, education, occupation, socio-economic status) and malaria preventive measures (bed net use), was conducted two weeks prior to the survey. Each house was identified with a unique number and geo-referenced using a handheld Global Positioning System (GPS) device (Garmin’s GPSMAP 60CSx, Garmin International Inc., USA).

The survey was done in November 2012. Each household was visited and all available children between six months and seven years old, plus one randomly selected individual above 7, were enrolled after providing written informed consent/assent. Whenever selected individuals were absent, the household was revisited within the next two days to maximize subject participation. Each participant had the axillary temperature taken, malaria symptoms were recorded, and a finger-prick blood sample collected for immediate microscopy (thick and thin blood smears) and on filter paper (Whatman grade 3, Whatman, Springfield Mill, USA) for later serological and molecular tests. Filter paper dried blood samples were individually stored at 4°C with desiccant until processed at Institute of Tropical Medicine Alexander von Humboldt, Lima (ITM-AvH). All individuals with a malaria infection detected by LM were treated according to the national guidelines.

### Laboratory procedures

#### Microscopy

Thick and thin smears were stained for 10 minutes with a 10% Giemsa solution, and parasite density was computed after counting the number of asexual parasites for 200 white blood cells (WBC) in the thick smear and assuming a concentration of 8,000 WBCs/μl. Slides were read during the survey and then a day later by an expert at microscopy at the reference laboratory in Iquitos. A slide was declared negative if no malaria parasite was found after examining 100 fields. Quality control was done blindly on all positive slides and 10% of randomly chosen negative slides by a senior technician at ITM-AvH. Any discordant results were reread by a second senior technician until agreement.

#### Species-specific Polymerase Chain Reaction (ss-PCR)

Parasite DNA was extracted using the Saponin lysis Chelex 100 method [27] and stored at 4°C for immediate use in PCR reactions or at -20°C for later use. All samples were first analyzed by ssPCR following a protocol published elsewhere [28]. Briefly, this ssPCR consisted of a primary PCR with primers directed to specific Plasmodium sequences, and obtained amplicons were used for a second PCR using *P*. *falciparum* and *P*. *vivax* specific primers.

#### Serology

Antibodies for specific IgG responses to *P*. *vivax* merozoite surface protein-1(19-kDa C-terminal region, PvMSP-1_19_) [29] and apical membrane antigen-1 (PvAMA-1) [30], and to *P*. *falciparum* glutamate-rich protein-fragment R2 (PfGLURP-R2) [31] and apical membrane antigen-1 (PfAMA-1) [32] were detected using ELISA. Although data on the kinetics of antibody responses are limited, some studies using similar antigens (such as AMA-1 and MSP1-19) have reported that these antibodies are not short-lived (lasting from months to several years) and that the half-live of those responses can be influenced by differences in the background immunity of populations and in the level of malaria transmission [33]. According to the ELISA protocol [19], dried blood filter paper samples (5mm diameter disc/sample) were eluted overnight at 4°C in 2 ml of PBS-Blotto-Tween. Two hundred microliters of the eluate were added in duplicate to blocked-ELISA plates coated separately with each antigen. Pooled sera from five *P*. *vivax* and five *P*. *falciparum* infected patients, and from five control individuals without recent (negative PCR) and past history of malaria infection were diluted at 1:400 in PBS-Blotto, as positive and negative control respectively. Goat anti-human IgG (H+L) specific peroxidase (Merck Millipore, USA) diluted to 1:20,000 in PBS-Tween was used as conjugate and incubated for 1 h before development of the ELISA using 200μl ABTS substrate-chromogen solution. Optical Density (OD) values at 405 nm (Lector Multi-Modal Synergy H1, BioTek, USA), were corrected by subtracting the mean OD of the antigen negative control wells from the mean OD of the corresponding antigen containing wells. Analyses with duplicate OD values that differed by more than 1.5-fold were rejected and rerun. To ensure a standardization of the sample results across ELISA plates, the percent positivity (PP) of each specimen was calculated using the OD of the positive control serum as 100%. Quality control was done blindly at ITM-AvH on 5% of randomly chosen samples. The criterion for positivity was determined for each antigen of each species applying a mixture model to the PP data which assumed two inherent Gaussian distributions: a narrow distribution or seronegatives, and a broader distribution of seropositives. Cut-off value was calculated as the mean plus 3 standard deviations of the narrow distribution [21].

### Data analysis

Survey data were double entered and cross-checked in Excel (Microsoft Corp, USA), and data analysis was performed with R v.2.15 software (R Development Core Team, R Foundation for Statistical Computing, Austria). Baseline characteristics between sites were compared using the Chi-squared test. Species-specific parasite prevalence (by LM and by PCR) as well as seroprevalence were estimated overall and by site.

Uni- and multivariate adjusted analyses were performed using survey logistic regression to determine risk factors for species-specific malaria outcomes (i.e. malaria infection, and malaria exposure), adjusting for the following potential confounders: site, age, gender, out-door occupation, predominant material in walls and roof, availability of electricity, and past history of malaria. Malaria infection was defined as an individual with a positive PCR result, regardless of symptoms; while malaria exposure to any of the two species was defined as an individual with a positive serology to any of the two antigens for each species. Factors with p-values <0.1 for the likelihood ratio test in the univariate analysis were considered for inclusion in the multivariate adjusted model. Using the manual backward methods, final models retained all factors that were significantly associated with the malaria outcome (p <0.05). Interactions were systematically checked for up to order two. All analyses took into account the following survey design characteristics: community as strata, household as primary sampling unit, and corresponding sampling weights (w) for children <7 years (w = 1) and for individuals ≥7 years (w = 1/n, where n = number of individuals ≥7 years at household).

The QGIS software QGIS v.2.6 (QGIS developer team, Open Source Geospatial Foundation) was used to map all surveyed individuals [34] and the SaTScan software v.9.3 (M Kulldorff and Information Management Services Inc, USA) [35] to detect spatial clusters of individuals with malaria infection as well as with malaria exposure in each site, using the following characteristics: purely spatial analysis, Bernoulli probability model [36], latitude/longitude coordinates, report of most likely clusters with no geographical overlap of secondary clusters, Gini optimizer cluster collection, maximum spatial cluster size equal to 50% of total population. The main analysis was done without adjustment for covariates, and a secondary analysis included identified risk factors as covariates. SaTScan applies multiple circular windows across the study area, each circle representing a possible cluster. Clusters were assessed based on 999 Monte Carlo simulations to determine the probability of observed frequency of infected/exposed individuals being due to chance relative to expected frequency under the null hypothesis of no clustering. The null hypothesis was rejected if any resulting p-value of assessed clusters was <0.05 and the window with the maximum log likelihood ratio (LLR) was identified as the most likely cluster. The relative risk (RR) reported for each identified cluster was the estimated risk within the cluster divided by the estimated risk outside the cluster [36].

Seroprevalence was stratified into yearly age-decile groups and then analyzed using a reverse catalytic conversion model [20] which allowed the estimation of two parameters: *a) a seroconversion rate (SCR*, *λ)* or force of infection (FOI) defined as the annual rate at which individuals change from seronegative to seropositive); and *b) a seroreversion rate (SRR*, *ρ)* defined as the annual rate at which seropositive individuals revert to a seronegative state. The main assumptions of the model are the following [37]: all individuals spent their entire live in the community, migration is negligible, there are no individual differences in SCR between subjects of a same community, and the disease transmission in the community is at an endemic steady-state, i.e. SCR is assumed constant over time.

Since study communities were established in the early 1980s, the first analysis excluded individuals older than 30 years old (21.2% of total population) in order to comply with the first assumption mentioned above. The dataset of all sites was first analyzed using a reversible catalytic model that assumed a stable transmission throughout time estimating SCR and SRR for the study population up to age 30 years. Then, the specific data set from each site was analyzed using two reversible catalytic models, one that assumed a constant-SCR over time and another that assumed a change in SCR at a given time point. All these models included the population SRR as common parameter. Profile likelihood plots were applied to determine the most likely time point for change in the transmission prior to survey [21]. The latter was subsequently incorporated into the catalytic model to fit a seroprevalence curve with two SCRs (before and after time cut-off) [22], which was then compared with a single SCR seroprevalence curve using the likelihood ratio (LR) test (p<0.05). Bootstrapped 95% confidence intervals for SCR and SRR were estimated using the bias-corrected and accelerated (BCa) bootstrap resampling method [38]. Later, a sensitivity analysis was done including individuals of all ages in order to assess the influence of the individuals older than 30 years old on the age-seroprevalence curves and on the identification of most likely time points for change in the transmission according to the model.

Additionally, routine surveillance data, namely reported clinical malaria episodes through PCD provided by the Regional Direction of Health Loreto, were used to explore the association between malaria incidence trends in the department (between 1990 and 2013), [2] and the changes in malaria transmission obtained from catalytic models. Since routine malaria surveillance data since 2002 could be reliably disaggregated only to the district level but not to the village level, an retrospective revision of all registered individuals detected by PCD between January and December 2012 was done in all health facilities near the study communities in order to estimate the annual parasite index for *P*. *vivax* (API*v*) and *P*. *falciparum* (API*f*) per site in the year of the survey.

### Ethical issues

Ethical clearance was obtained from the Ethics Review Board of the Universidad Peruana Cayetano Heredia, Lima, Peru (SIDISI code: 059745). Permissions were received from health and local authorities after explaining the purpose and procedures of the study. Signed informed consent was obtained prior to participation and blood sampling by all adults and the parents of all participating children <18 years. Besides their parent's consent, children older than 7 years provided a signed informed assent.

## Results

**Fig 2:**
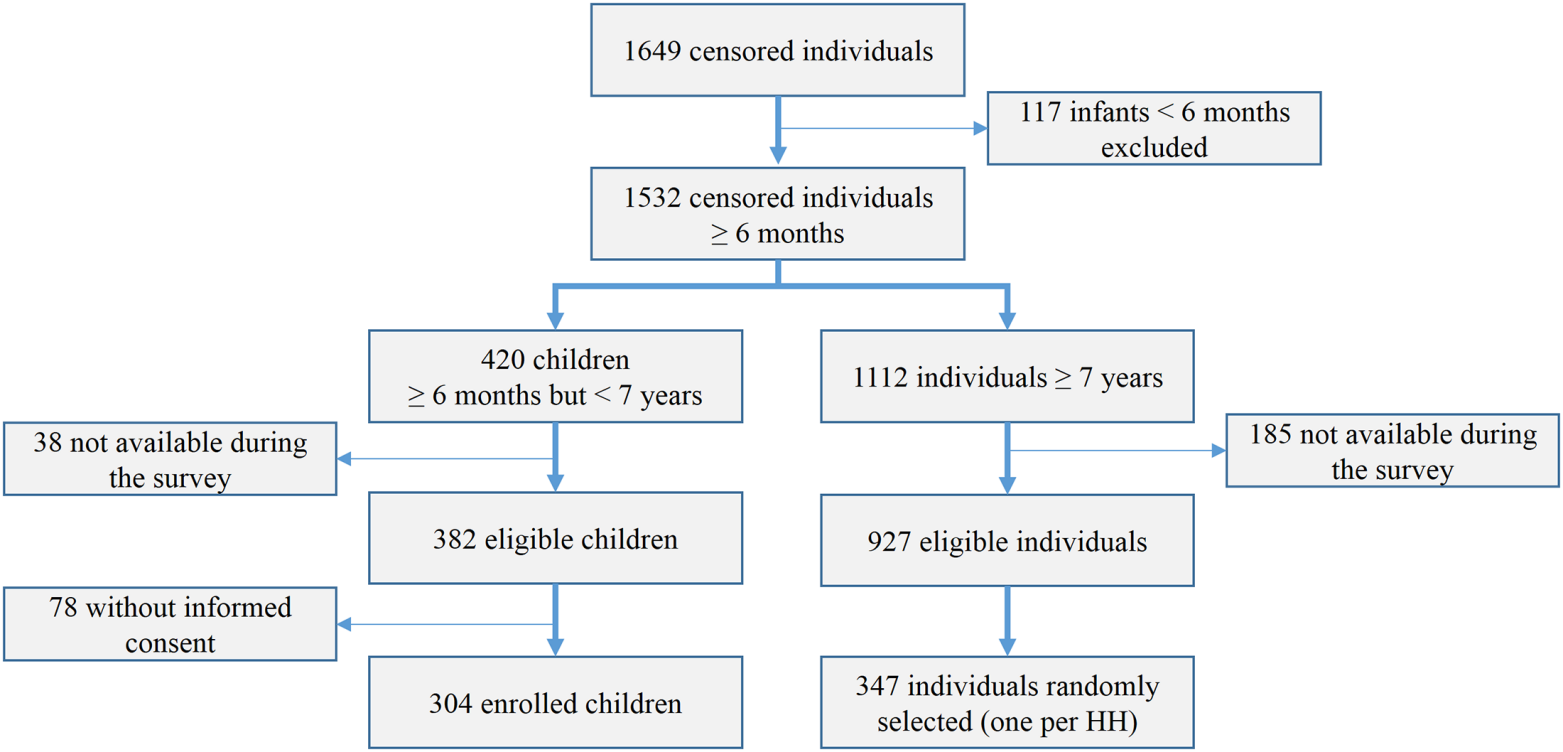
Flowchart of study participants.

A total of 348 houses and 1,649 individuals were identified during the census, with the following site specific distribution: Site A = 129 houses (H) and 655 individuals (Ind); Site B = 80 H and 377 Ind; Site C = 139 H and 617 Ind. The overall ratio of female to male was 1.04; half of the population was less than 15 years old. The survey enrolled 651 individuals: 304 (72.4%) of the total 420 censored children between 6 months and 7 years, and 347 (31.2%) individuals of the total individuals ≥ 7 years. Fig 2 presents the flowchart of participants and S1 Table shows the survey coverage by site and age group. Of the total 651 individuals, 238 (36.6%) lived in Site A, 153 (23.5%) in Site B, and 260 (39.9%) in Site C (Table 1). Children under 7 represented between 45% and 48% of the participants across sites, and females slightly outnumbered males in site A (ratio female/male = 1.15; p = 0.27). The majority of adult participants had only primary school education level, except in site A where 70.5% had secondary or higher education level (p<0.001). Overall, the most commonly reported occupation among individuals aged ≥15 years was housewife, followed by farmers and guards in sites B and C, while activities were more diversified including traders and indoor laborers in Site A (p<0.001). In all sites, most of houses had wooden walls, with roofs of predominantly palm leaf and straw in Sites B and C, and tin sheets in Site A (p<0.001). Reported bed net use was very high (>95%) in all sites; nevertheless, most participants in site A stated of never have experienced malaria, while this was not the case in the other two sites (p<0.001).

**Table 1:** Baseline characteristics of the study population.

	Site A		Site B		Site C		Total	
	(AG, AB, PC)		(SC, BP, EM)		(ET, SL)			
	n	%	n	%	n	%	N	%
**Total surveyed**	238	36.6	153	23.5	260	39.9	651	100.0
**Gender**								
Female	137	57.6	77	50.3	134	51.5	348	53.5
**Age groups (years)**								
< 7	109	45.8	74	48.4	121	46.5	304	46.7
7–14.9	42	17.6	19	12.4	40	15.4	101	15.5
15–30	47	19.7	17	11.1	44	16.9	108	16.6
> 30	40	16.8	43	28.1	55	21.2	138	21.2
**Education (> = 18 years, n = 218)** *								
None	0	0.0	2	3.6	3	3.5	5	2.3
Primary	23	29.5	28	50.9	55	64.7	106	48.6
Incomplete secondary	29	37.2	11	20.0	18	21.2	58	26.6
Complete secondary/+	26	33.3	14	25.5	9	10.6	49	22.5
**Occupation (≥15 years, n = 246)** *								
None (students, retired)	12	14.1	7	11.7	12	11.9	31	12.6
Housewife	46	54.1	23	38.3	41	40.6	110	44.7
Laborer/trader/mototaxi driver	22	25.8	3	5.0	2	2.0	27	11.0
Farmer/guard/logger/fisher	5	5.9	27	45.0	46	45.5	78	31.7
**Predominant material in roof** *								
Palm leaf, straw	56	22.8	88	56.0	197	79.9	341	51.5
Tin roof	182	77.2	65	44.0	56	20.1	303	48.5
Missing					7		7	
**Electricity availability** *								
Yes	225	96.0	91	65.3	113	47.6	429	65.9
**Sleeping under bednet the previous night**								
Yes	227	96.7	149	99.7	259	100.0	635	98.8
Missing	4		3		1		8	
**Self-report of confirmed malaria episode** *								
Never	182	65.0	74	31.3	135	42.7	391	60.4
In the past 12 months	7	3.8	25	19.1	66	29.5	98	15.1
>12 months	48	31.1	54	49.6	56	27.8	158	24.4
Missing	1				3		4	

* p-value <0.05 for differences between sites

By LM, the overall parasite prevalence was 3.3% (7 *P*. *vivax* and 10 *P*. *falciparum* infections), and by PCR 10.9% (18 *P*. *vivax*, 34 *P*. *falciparum* and one mixed infections) (Table 2), with the highest figures in the two rural sites B and C. Species-specific parasite prevalence varied widely across the sites by both LM and PCR (p<0.001). By PCR, both species had similar prevalence in site C (8.0%) and A (3.0 *vs* 1.1%), while in site B *P*. *falciparum* was predominant (11.2%) over *P*. *vivax* (2.1%). The species specific seroprevalence showed similar heterogeneity between sites. *P*. *vivax* seroprevalence was equally high in sites B (42.3%) and C (46.7%), and relatively low in site A (16.4%) (p<0.001). *P*. *falciparum* seroprevalence was 36.7% in site B, 16.7% in site A and 18.7% in site C (p<0.001). Interestingly for both species, sites with the highest seroprevalence were respectively the ones with the highest prevalence by PCR, and also the ones with the highest API estimated from PCD records in 2012. Gametocytes were found in 4 (44.4%) of the total 9 microscopically confirmed *P*. *falciparum* infections in site B, as well as in 4 (57.1%) of the total 7 microscopically confirmed *P*. *vivax* infections in site C. The unique microscopically confirmed *P*. *falciparum* infection in site C had also gametocytes. Most of PCR-positive infections were sub-microscopic (>70%) and asymptomatic (>75%) at the time of the survey, regardless of the *Plasmodium* species (S2 Table). Species-specific PCR positivity was significantly associated with species-specific seropositivity both overall and for each specific antigen (p < 0.05).

**Table 2:** Malaria prevalence (by microscopy and PCR) and seroprevalence by study site.

	Site A			Site B			Site C			Total		
	n	%	95% CI	n	%	95% CI	n	%	95% CI	n	%	95% CI
**Microscopy**												
*P*.*vivax* *	0	0.0		0	0.0		7	4.9	[2.1,10.8]	7	1.8	[0.8,4.1]
*P*.*falciparum* *	0	0.0		9	6.4	[3.0,13.1]	1	0.2	[0.0,1.2]	10	1.5	[0.7,3.1]
Total*	0	0.0		9	6.4	[3.0,13.1]	8	5.0	[2.2,10.9]	17	3.3	[1.9,5.8]
**PCR**												
*P*.*vivax* *	1	1.1	[0.1,7.3]	2	2.1	[0.5,8.1]	15	8.0	[4.4,14.0]	18	3.9	[2.3,6.5]
*P*.*falciparum* *	6	3.0	[1.2,7.8]	14	11.2	[6.2,19.2]	14	8.0	[4.2,14.6]	34	6.7	[4.5,9.9]
Mixed	0	0.0		1	1.1	[0.1,7.2]	0	0.0		1	0.2	[0.0,1.7]
Total	7	4.1	[1.7,9.6]	17	14.4	[8.6,23.0]	29	15.9	[10.5,23.5]	53	10.9	[8.0,14.6]
**Seropositivity *P*.*vivax***												
MSP1-19*	13	8.1	[4.2,14.8]	26	24.2	[16.5,34.0]	88	38.5	[30.6,47.1]	127	23.1	[19.2,27.6]
AMA-1*	18	11.9	[7.0,19.5]	35	33.5	[24.5,43.9]	67	34.5	[27.1,42.6]	120	25.2	[21.1,29.9]
Total*	27	16.4	[10.7,24.4]	45	42.3	[32.6,52.6]	102	46.7	[38.5,55.1]	174	33.6	[29.0,38.5]
**Seropositivity *P*. *falciparum***												
GLURP*	26	13.7	[8.8,20.8]	34	27.9	[19.9,37.7]	39	15.8	[10.8,22.5]	99	17.7	[14.2,21.8]
AMA-1*	9	4.3	[1.9,9.2]	22	20.7	[14.0,29.7]	18	8.5	[4.9,14.6]	49	9.6	[7.1,12.8]
Total*	33	16.7	[11.5,23.8]	46	36.7	[27.9,46.5]	47	18.7	[13.2,25.8]	126	22.0	[18.3,26.3]
**Incidence rate (2012)**												
API*v* (x1000)^+^		7.6			66.3			205.8				
API*f* (x1000)^+^		4.6			148.5			14.6				
API *total* (x1000)^+^		12.2			214.8			220.4				

*p-value<0.05 for significant differences between sites.

^**+**^ API*v* = Annual parasite index for *P*. *vivax*, API*f* = Annual parasite index for *P*. *falciparum*, API*tot*a*l* = Annual parasite index

Given the small number of PCR-positive infections by species, multivariate adjusted risk factor analysis was carried out only for any parasite infection. Only site and age remained independently associated with malaria infection: while individuals living in Site B (AOR: 3.9, 95% CI [1.4–11.5]) and in Site C (AOR: 4.5, 95% CI [1.6–12.7]) were more likely to be infected than those living in Site A, individuals >30 years had higher odds of malaria infection than children < 7 years (AOR: 2.4, 95% CI [1.0–5.7]). Considering *P*. *vivax* exposure, the multivariate adjusted risk factor analysis showed that site and age remained independently associated with the *P*. *vivax* seropositivity: individuals living in Site B (AOR: 3.9, 95% CI [2.0–7.7]) and in Site C (AOR: 5.1, 95% CI [2.8–9.3]) had higher odds of *P*. *vivax* seropositivity than those in Site A, and those older than seven years had increasingly higher odds (test for trend p < 0.001) of *P*. *vivax* seropositivity compared to younger children (Table 3). For *P*. *falciparum* exposure, site, age and outdoor occupation remained independent risk factors in the final model: individuals living in Site B (AOR: 2.3, 95% CI [1.2–4.4]) were more likely to be exposed to *P*. *falciparum* than those in Site A, adults >30 years (AOR: 2.1, 95% CI [1.1–4.0]) were more exposed than children < 7 years, and people working outdoor were three times more exposed to *P*. *falciparum* (AOR: 3.1, 95% CI [1.5–6.6]). Despite being significantly associated with both *P*. *vivax* (OR: 6.8, 95% CI [3.7–12.5]) and *P*. *falciparum* exposure (OR: 2.7, 95% CI [1.5–4.8]) in the univariate analysis, the history of malaria in the past year was not included in the multivariate analysis, since it is not a risk factor for malaria infections per se but rather a consequence of previous exposure to given risk factors.

**Table 3:** Univariate and multivariate risk factor analysis for species-specific malaria exposure.

	Univariate						Multivariate	
	*P*.*vivax*			*P*.*falciparum*			*P*.*vivax*	*P*.*falciparum*
	%	n/N	OR [95% CI]	%	n/N	OR [95% CI]	AOR [95% CI]	AOR [95% CI]
**Site**								
A	16.4	27/238	1	16.7	33/238	1	1	1
B	42.3	45/152	3.7* [2;7.1]	36.7	46/152	2.9* [1.6;5.2]	3.9* [2;7.7]	2.3* [1.2;4.4]
C	45.0	102/259	4.4* [2.4;8.1]	18.7	47/259	1.1 [0.6;2.1]	5.1* [2.8;9.3]	0.8 [0.4;1.6]
**Age groups (years)**								
< 7	13.1	40/303	1	13.7	39/303	1	1	1
7–14.9	27.0	27/101	2.5* [1.5;4.2]	18.0	16/101	1.4* [0.7;2.6]	2.9* [1.7;5]	1.5 [0.8;2.8]
15–30	34.8	35/107	3.6* [2.1;6.1]	18.3	20/107	1.4* [0.7;2.7]	4.3* [2.4;7.7]	1.3 [0.7;2.6]
> 30	52.6	72/138	7.4* [4.5;12]	34.7	51/138	3.3* [1.9;5.7]	8.8* [5.2;14.8]	2.1* [1.1;4]
**Gender**								
Female	29.6	80/346	1	16.9	54/346	1		
Male	38.7	94/303	1.5° [1;2.3]	28.6	72/303	2* [1.2;3.2]		
**Outdoor occupation (farmer, guard, logger, fisher)**								
No	29.0	127/571	1	18.1	91/571	1		1
Yes	63.3	47/78	4.2* [2.4;7.6]	47.5	35/78	4.1* [2.3;7.5]		3.1* [1.5;6.6]
**Predominant material in roof**								
Palm leaf, straw	25.1	52/302	1	19.0	43/302	1		
Tin roof	41.5	120/340	2.1* [1.3;3.4]	24.6	82/340	1.4 [0.8;2.3]		
**Electricity availability**								
Yes	29.3	95/428	1	20.9	76/428	1		
No	44.1	79/221	1.9* [1.2;3.0]	24.7	50/221	1.2 [0.7;2.1]		
**Malaria episode in the past 12 months**								
No	26.3	108/547	1	18.9	89/547	1		
Yes	70.8	66/98	6.8* [3.7;12.5]	38.3	37/98	2.7* [1.5;4.8]		

*p<0.05

° p<0.1

**Fig 3:**
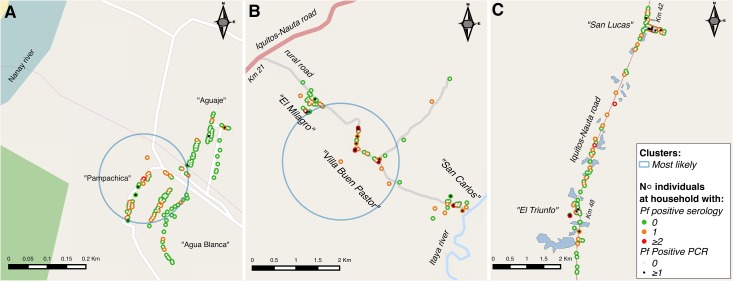
Distribution of *P*. *falciparum* infected and exposed individuals by study site (A, B, C) with respective significant spatial clusters for *P*. *falciparum* seropositivity.

**Fig 4:**
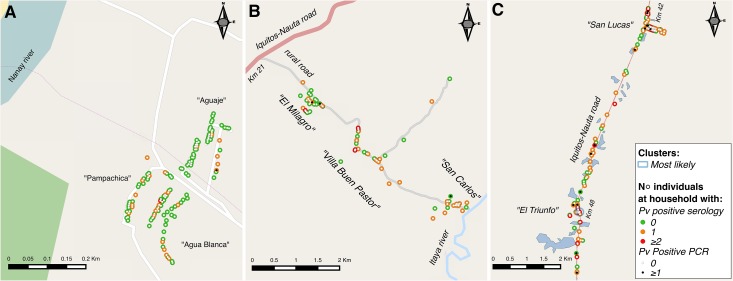
Distribution of *P*. *vivax* infected and exposed individuals by study site (A, B, C) with respective significant spatial clusters for *P*. *vivax* seropositivity.

Figs 3 and 4 show the spatial distribution of infected (positive PCR) and exposed (positive serology) individuals by species and for each site. Despite some aggregation of cases within adjacent houses in sites B and C, no significant spatial clusters of infections were identified by SaTScan, mainly because of the low number of infections. However, the purely spatial analysis without adjustment for covariates confirmed that malaria exposure mainly to *P*. *falciparum* was not randomly distributed. In site A, the most likely spatial cluster of *P*. *falciparum* seropositive individuals (RR = 6.3, p<0.001), located on the western part of the village close to Nanay river (Fig 3A), had a 120m radius area which included 114 surveyed individuals (47.9% of total) in 59 households (45.7% of total). This cluster represented 85.3% (29/34) of all *P*. *falciparum* seropositive individuals, but only two of the six PCR-confirmed infections by species. In site B, the most likely *P*. *falciparum-*exposure cluster (RR = 2.9, p = 0.001) had a radius of 1.3 km including 50 individuals (32.7% of the total) in 30 households (37.5% of the total) (Fig 3B). This cluster represented 58.7% (27/46) of all 46 *P*. *falciparum* seropositive individuals in the site, and 9 (64.2%) of the 14 *P*. *falciparum* mono-infections detected by PCR. Site C had no significant spatial cluster of *P*. *falciparum* exposure but was the only site where a most likely cluster of *P*. *vivax*-exposure (RR = 2.9, p = 0.001) could be identified (Fig 4C). This was a small cluster of 68m radius that included 10 households, all surveyed individuals were *P*. *vivax* seropositive but none of them was infected (PCR negative). Adding age group and/or outdoor occupation (identified in the risk factor analysis) as covariates in SaTScan analysis to locate species-specific exposure clusters had no effect on results.

**Fig 5:**
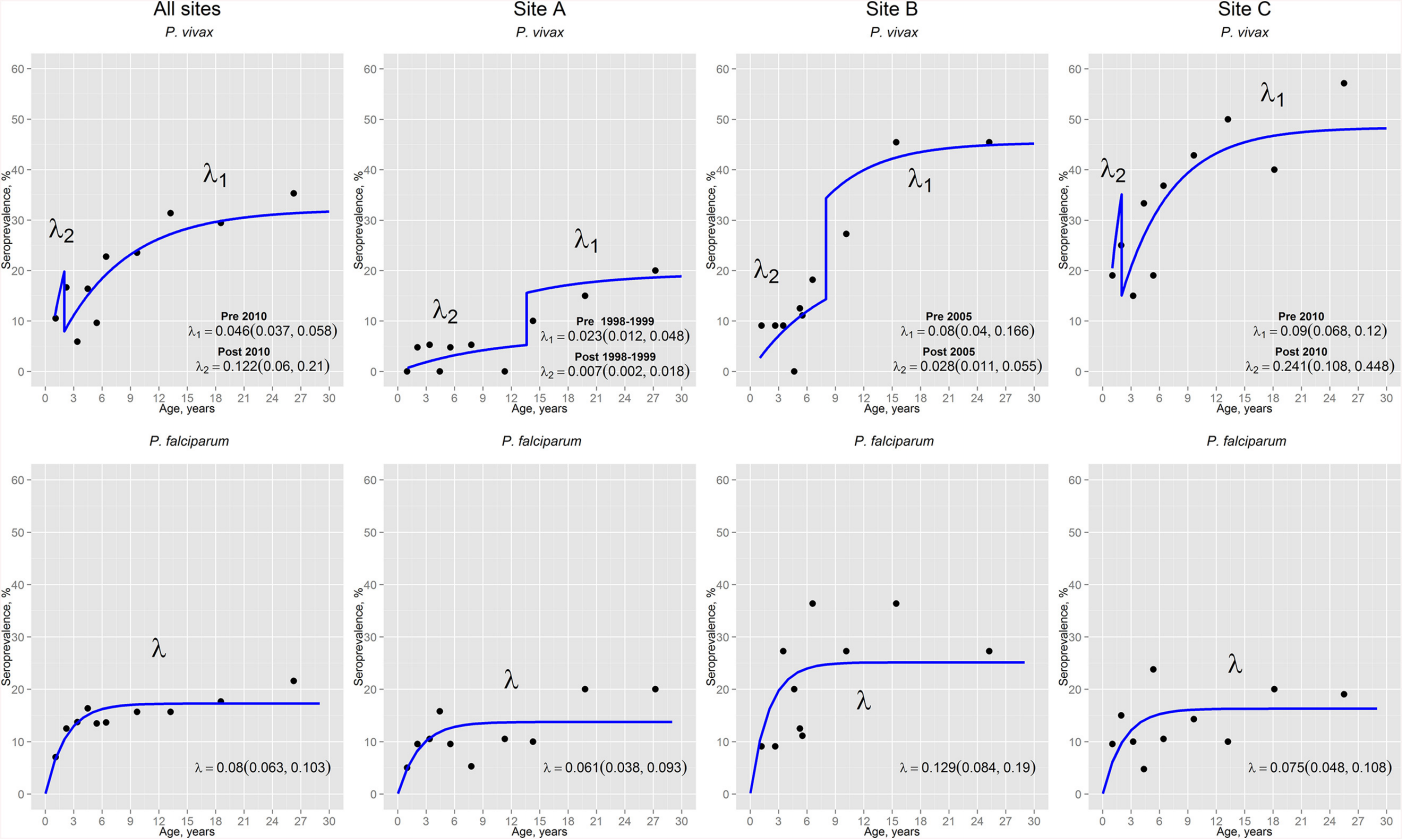
*P*. *vivax* and *P*. *falciparum* age-seroprevalence curves for individuals under 30 years old in each site. Black dots represent observed prevalence, whilst the blue lines represent the maximum likelihood model. Seroconversion rates (λ, (95%CI)) are plotted on the graphs. Two seroconversion rates (λ_1_ and λ_2_) are plotted in graphs if likelihood ratio tests indicate change at a certain point in calendar time.

Age seroprevalence curves of individuals aged ≤ 30 years confirmed a significant increase of malaria seroprevalence with age, but with different species specific patterns (Fig 5). The overall (all sites) *P*. *falciparum* age seroprevalence was better described by a model with only one SCR (λ = 0.08) and one SRR (ρ = 0.384), while the overall *P*. *vivax* age seroprevalence was best modeled with two SCR (pre-2010-λ1 = 0.046, post-2010- λ2 = 0.122) and one SRR (ρ = 0.096) indicating an increase in MTI in 2010 for this species (LRT, p = 0.01). The reversible catalytic models for *P*. *falciparum* showed a stable transmission in each study site, with higher transmission intensity in Site B (λ = 0.129) than in sites A (λ = 0.061) and C (λ = 0.075). Conversely, best models for *P*. *vivax* differed by site. While site C had a change in *P*. *vivax* MTI in 2010 with a significant threefold increase (pre-2010 λ1 = 0.09, post-2010 λ2 = 0.241, p = 0.02), sites A and B experienced significant drops in SCR but at different times: 13–14 years before the survey in site A (pre-1998-1999 λ1 = 0.023, post-1998-1999 λ2 = 0.007, p = 0.04), and 7–8 years prior survey in site B (pre-2005 λ1 = 0.028, post-2005 λ2 = 0.008, p = 0.03). Moreover for both species, sites with the highest SCRs (SCR after the time cut-off in models with two SCRs) were respectively the ones with the highest API.

Although the inclusion of seroprevalence data of individuals aged >30 years did not change the assumption of a stable transmission over time for *P*. *falciparum* per site (site A, λ = 0.024; site B, λ = 0.063; site C, λ = 0.033), it allowed identifying a decrease in SCR for *P*. *falciparum* 32 years before the survey when the analysis included all the sites (pre-1980 λ1 = 0.05, post-1980 λ2 = 0.031, p = 0.03) (S1 Fig). For *P*. *vivax*, the incorporation of antibody responses of individuals >30 years had different effects. While in all sites and site C it was identified an increase in SCR for *P*. *vivax* two years before the survey (all sites: pre-2010 λ1 = 0.035, post-2010 λ2 = 0.117, p = 0.002; site C: pre-2010 λ1 = 0.061, post-2010 λ2 = 0.231, p = 0.003), *P*. *vivax* seroprevalences curves in site A was best described by a model assuming a decrease in malaria transmission 37 years prior survey (pre-1975 λ1 = 0.031, post-1980 λ2 = 0.009, p = 0.008). Conversely, model results for site B indicated a constant *P*. *vivax* SCR over time (λ = 0.04).

**Fig 6:**
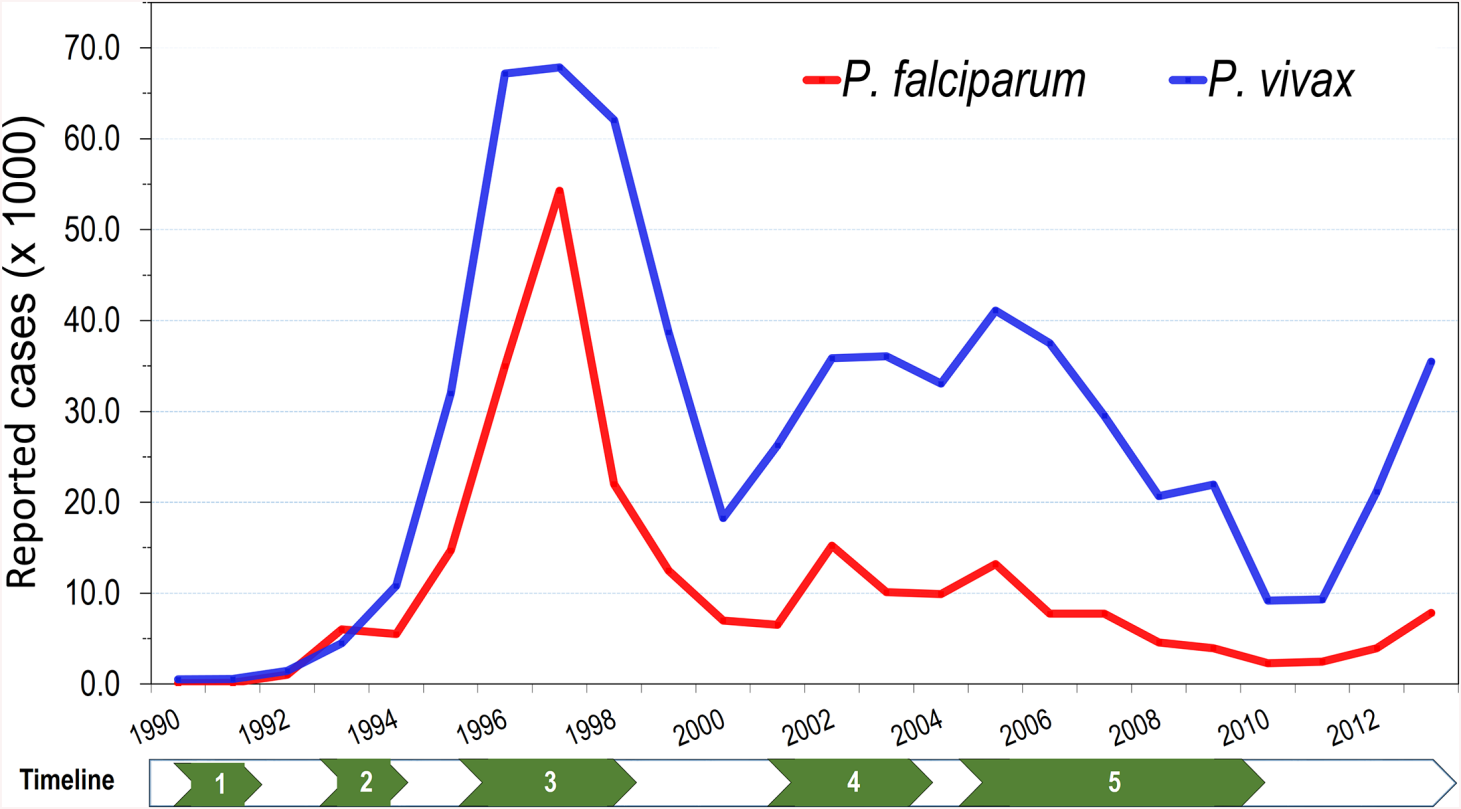
Evolution of the annual malaria incidence in Loreto department: 1990–2013. Information on annual malaria cases was obtained from the Regional Health Direction of Loreto. Numbers in the timeline point out important events that influenced malaria incidence: 1) First report P. falciparum in Loreto (1990), 2) first reports of chloroquine resistance (CQR) in P. falciparum (since 1994), 3) very strong ENSO phenomenon (1996–1998), 4) Implementation of new antimalarial treatment policy for P. falciparum and P. vivax (2001–2004), 5) Global fund-PAMAFRO project (2005–2010).

The retrospective analysis (1990–2013) of annual malaria incidence in Loreto department (Fig 6) showed a peak during the malaria epidemic (1996–1998) associated with the ENSO phenomenon when more than 65,000 *P*. *vivax* and 50,000 *P*. *falciparum* cases were recorded. After the epidemic, incidence dropped drastically and stabilized between 2000 and 2001 at around 30,000 total annual cases, and at 45,000 and 55,000 cases between 2002 and 2005. During this post-epidemic period, a new antimalarial treatment policy (2001–2004) was progressively implemented as previously described. During the Global Fund-PAMAFRO Project (2005–2010), intense control efforts resulted in a marked decline of malaria incidence from 54,291 reported clinical cases in 2005 to 11,604 cases in 2010. However, since 2011 (11,703 cases), malaria incidence is doubling each year. Noteworthy, *P*. *falciparum* infections usually followed similar trends than *P*. *vivax*, accounting for 20–25% of the total malaria cases, with exception of 1996–1998 when they represented almost 45% of the cases in Loreto. Between 2002 and 2013, though San Juan and Iquitos districts followed similar trends of malaria incidence than Loreto department (S2 Fig), *P*. *falciparum* cases started to increase two years earlier (2009) in these two districts.

## Discussion

The combined analysis of parasitological and serological measures collected during a single cross-sectional survey allowed for an in-depth characterization of the malaria transmission patterns in three different sites in an endemic district of the Peruvian Amazon. Most malaria infections detected by PCR were sub-microscopic and asymptomatic. The site specific analysis of both prevalence and seroprevalence data showed a high degree of heterogeneity at micro-geographic level and in time for both *P*. *falciparum* and *P*. *vivax*. However, species-specific parasitological and serological metrics (i.e., seroprevalence, SCRs) were in good agreement with IPA estimates by species.

Although the survey was conducted in November, before the usual peak of clinical malaria cases, an important number of malaria-infected individuals were identified, mainly by *P*. *falciparum*, and most of them were undetectable by microscopy and free of malaria-related symptoms. As reported by other countries in the region, asymptomatic malaria infections (including submicroscopic infections) are common in low endemic settings, representing a “hidden” human reservoir likely to contribute to maintain transmission [39–42]. Previous cross-sectional studies using PCR in rural peri-Iquitos communities have also reported high prevalence of asymptomatic parasite carriers (5%-14%)[7,8,43]; however, the surveys were conducted at the time of high seasonal transmission and *P*. *vivax* was always the predominant species.

Although routine surveillance data at district and department level showed a predominance of *P*. *vivax* over *P*. *falciparum* reported cases, parasitological and serological measures indicated that species-specific malaria transmission may vary widely across sites. Indeed, site C had similar prevalence of both species by PCR, but the higher seroprevalence and SCR estimates for *P*. *vivax* compared to those of *P*. *falciparum* suggested a very recent increase in *P*. *falciparum* infections. Moreover, in site B the higher *P*. *falciparum* seroprevalence and SCR compared to those of *P*. *vivax* suggested that the predominance of *P*. *falciparum* infections detected by PCR was not new. Additionally, the identification of *P*. *falciparum* gametocyte carriers by microscopy suggested ongoing transmission of this species in the site. These hypothesis were further supported by the newly generated site incidence data on *P*. *falciparum* (IPA*f*) and *P*. *vivax* (IPA*v*) in 2012. These results highlight the added value of combining parasitological and serological metrics in accurately understanding the local epidemiology since data from the health information system are often missing or not very reliable.

The differences in species-specific seroprevalence across sites were in line with findings from previous entomological studies, all reporting a high heterogeneity in malaria transmission in the peri-Iquitos region [27,44,45]. During the late 90s malaria epidemic, EIRs for *P*. *falciparum* were significantly higher in rural communities along the Iquitos-Nauta road than in periurban areas, with large monthly variations ranging from 0 to 12 infective bites per person per month [45]. More recent studies have shown a positive association between EIR and level of deforestation [44] as well as significant heterogeneity between collection sites and over time, reaching sometimes comparable EIR estimates to holoendemic African areas [27]. Deforestation in areas of site B and C following road construction and subsequent population settlements may have resulted in favorable ecological conditions for mosquito breeding and resting sites near households. Indeed, previous reports showed that natural water bodies and fish ponds (such those located in sites B and C) constituted ideal breeding sites for *An*. *darlingi*, and were significantly associated with malaria infection [44,46]; while surrounding secondary vegetation in previously deforested zones would provide resting place for adult mosquito populations [44]. Besides ecological factors, human socio-economic differences such as poorer housing conditions (mostly semi-closed houses) and higher proportion of individuals with outdoor economic activities in rural sites B and C compared to site A, may play an important role in increasing exposure to mosquito bites [47,48].

Genetic factors may play a role in human susceptibility to malaria infection and human infectiousness to mosquitoes [49,50]. However, there is insufficient data to establish their role in determining heterogeneity of malaria transmission in the Peruvian Amazon. The later could be partly due to the biological differences between *P*. *falciparum* and *P*. *vivax* [51,52]. Even though in theory *P*. *vivax* relapse patterns may influence serological profiles, the quantification of their effect on the production of malaria antibodies remains unclear due to the difficulty to classify a recurrent parasitaemia as relapse, new infection or recrudescence [51,52].

Species specific heterogeneity was not only observed at small-scale geographical level but also over time as illustrated by the respective SCR estimates calculated by models including and excluding individuals older than 30 years old. A strong advantage of SCRs is the ability to reconstruct the history of malaria exposure in a population [12], but one important assumption of the model is that all individuals in a specific community have experienced the same disease transmission intensity over time. Thus, reductions in malaria transmission identified more than 30 years ago, as identified by catalytic models using seroprevalence data of all ages, may be explained by higher exposure to malaria of this age group prior to the settlement in 1980s of the study communities.

On the other hand, SCR estimates generated using seroprevalence data of individuals aged ≤ 30 years would be more accurate to identify more recent changes in malaria transmission. Indeed, reversible catalytic models used to generate *P*. *vivax* seroprevalence curves showed that the significant decline in malaria transmission in sites A and B occurred at different points in time. While in site A, this time point coincided with the drastic reduction in *P*. *vivax* incidence after the late 90s epidemic; in site B, the change was concordant with the start in 2005 of the Global Fund-PAMAFRO project which prioritized comprehensive malaria control interventions in rural communities [5,6,53]. Since the end of this project in 2011, malaria incidence has steadily increased and already reached pre-PAMAFRO levels in 2013 and 2014, highlighting the potential risk of rapid malaria resurgence in the Amazon Region [2]. This was captured by the age-seroprevalence model for *P*. *vivax* in site C, i.e. increase in MTI two years before the survey. The fact that the catalytic model did not detect similar changes in SCRs for *P*. *falciparum* transmission as for *P*. *vivax*, could be due to the lack of power due to the lower levels seropositivity for *P*. *falciparum* and therefore insufficient sample size. Moreover, the independent association of outdoor economic activities with *P*. *falciparum* exposure would influence the model fit to age-seroprevalence data, since the assumption of the catalytic model that *P*. *falciparum* transmission is homogeneous in the community would not be fulfilled [37].

Effective methods for quantifying micro-geographical variations in malaria exposure should be able to identify stable hotspots of transmission over time [10]. The spatial analysis of cumulative clinical malaria incidence over several seasons should be a good method for detecting hotspots; provided most malaria infections are symptomatic, detected by health facilities, and accurately traced for place of infection and/or residence. However, this is rarely the case in most endemic settings, especially in low endemic area such as the one in our study, where most infections remain asymptomatic. Therefore the spatial identification of hotspots using serological measures may produce more robust results [17,54]. Serological markers can also be used to identify high risk sub-groups (or “hot-pops”)[55] within communities that could be targeted by additional interventions such as focal screening and treatment (FSAT) or mass drug administration (MDA)[56], which are financially and logistically unattractive at when implemented at large scale.

Our spatial analysis using serological data allowed the identification of clusters with the highest *P*. *falciparum* exposure: in site A, this cluster covered the area closest to the river which floods every year during the rainy season; in site B, the cluster included mainly households from Villa Buen Pastor, the community closest to natural water bodies and surrounding secondary vegetation, and with most individuals having outdoor occupations (i.e. farmers and guards). The quasi absence of clustering in site C may be explained by the local mosquito and human mobility patterns. Indeed the presence of several permanent breeding sites (i.e. fish ponds) along the Iquitos-Nauta road could result into a wider dispersal of *An*. *darlingi* and numerous movements of infected individuals through the unique and highly accessible road could further increase the dispersal of parasites. Although a similar explanation could be advanced for the absence of *P*. *vivax* clusters in site A and B, it is difficult to know how the relapse patterns can affect the spatial analysis using serological data.

Our study has some limitations. First, despite the use of two antigens of proven sensitivity for each *Plasmodium species* [18,57], regional variability in immunogenicity cannot be excluded and could be overcome by combining more antigens [16,22]. Though further research is still needed to identify the best antigens for measuring MTI [33] in Peruvian malaria endemic areas, we believe that sets used in our study were adequate and complementary since the antibody response produced was a good proxy for malaria exposure in other south-American endemic areas [18,58,59]. Ongoing cohort studies in the Peruvian Amazon will contribute to provide insights into the kinetics of antibody responses in the region. Second, the assumption by the mixture model of a bi-modal distribution of seropositives and seronegatives for the determination of the cut-off for seropositivity may not be true. However, in the absence of a good “gold standard”, this approach is widely accepted for determining an event status such as “malaria exposure” [60]. Third, due to the lack of information about SRR in the Amazon region, we included a common species-specific SRR (the study population SRR) into each corresponding site model as previously reported in other serological studies [58]. Although this approach allows comparing SCR estimates across sites, it is important to note that marked differences in SRR across sites could influence significantly the SCR estimates. Finally, despite the characteristics of the survey sampling, an enough proportion of individuals of each age group were included in each study site reducing the potential for selection bias. However, detecting the time point for change in malaria transmission as well as estimating corresponding SCRs may be slightly less accurate before than after 7 years prior the survey.

## Conclusions

The use of a single parasitological and serological malaria survey has proven to be an efficient and accurate method to characterize the species specific heterogeneity in malaria transmission at micro-geographical level as well as to identify recent changes in transmission. This approach, once optimized by a validated set of best antigens by geographical area, represents a reliable and effective tool to accurately identify and characterize hotspots of transmission and consequently help decision-makers to better design and deliver targeted interventions. This is particularly important in low transmission settings moving towards malaria elimination such as the Peruvian Amazon.

## Supporting Information

S1 Fig *P*. *vivax* and *P*. *falciparum* age-seroprevalence curves for individuals of all ages in each site. Black dots represent observed prevalence, whilst the blue lines represent the maximum likelihood model. Seroconversion rates (λ, (95%CI)) are plotted on the graphs. Two seroconversion rates (λ1 and λ2) are plotted in graphs if likelihood ratio tests indicate change at a certain point in calendar time.
(PNG)

S2 Fig Evolution of the annual malaria incidence in San Juan/Iquitos districts: 2002–2013. (TIF)

S1 Table Survey coverage by site. (DOCX)

S2 Table Association between PCR results and microscopy, serology and symptoms. (DOCX)
