# Supplementary figures and images for: Hotspots of Malaria Transmission in the Peruvian Amazon: Rapid Assessment through a Parasitological and Serological Survey
Source: PLoS One. 2015 Sep 10;10(9):e0137458. doi: 10.1371/journal.pone.0137458 (PMC4565712; doi:10.1371/journal.pone.0137458)

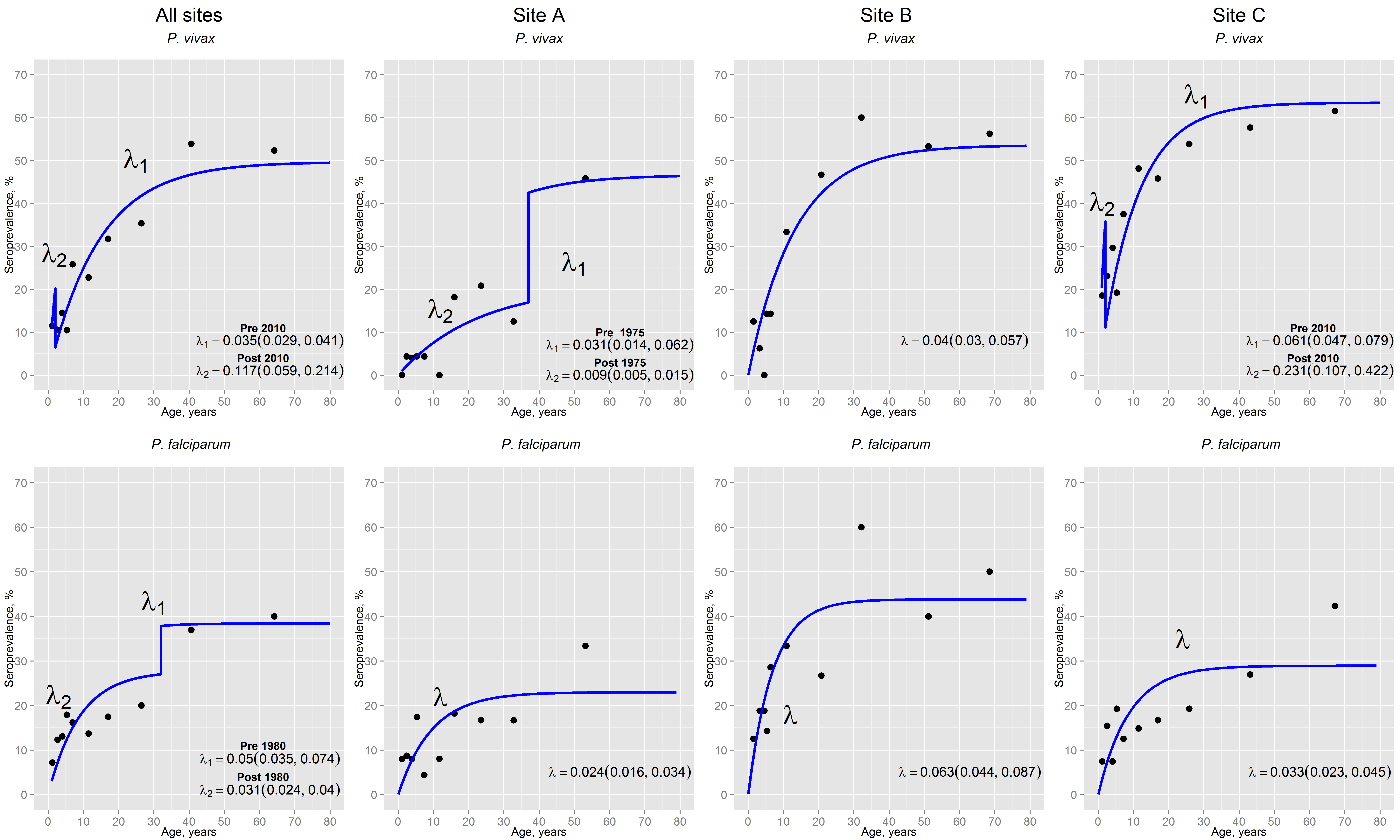

Supplement: S1 Fig — Black dots represent observed prevalence, whilst the blue lines represent the maximum likelihood model. Seroconversion rates (λ, (95%CI)) are plotted on the graphs. Two seroconversion rates (λ1 and λ2) are plotted in graphs if likelihood ratio tests indicate change at a certain point in calendar time. (PNG) [file pone.0137458.s001.png]

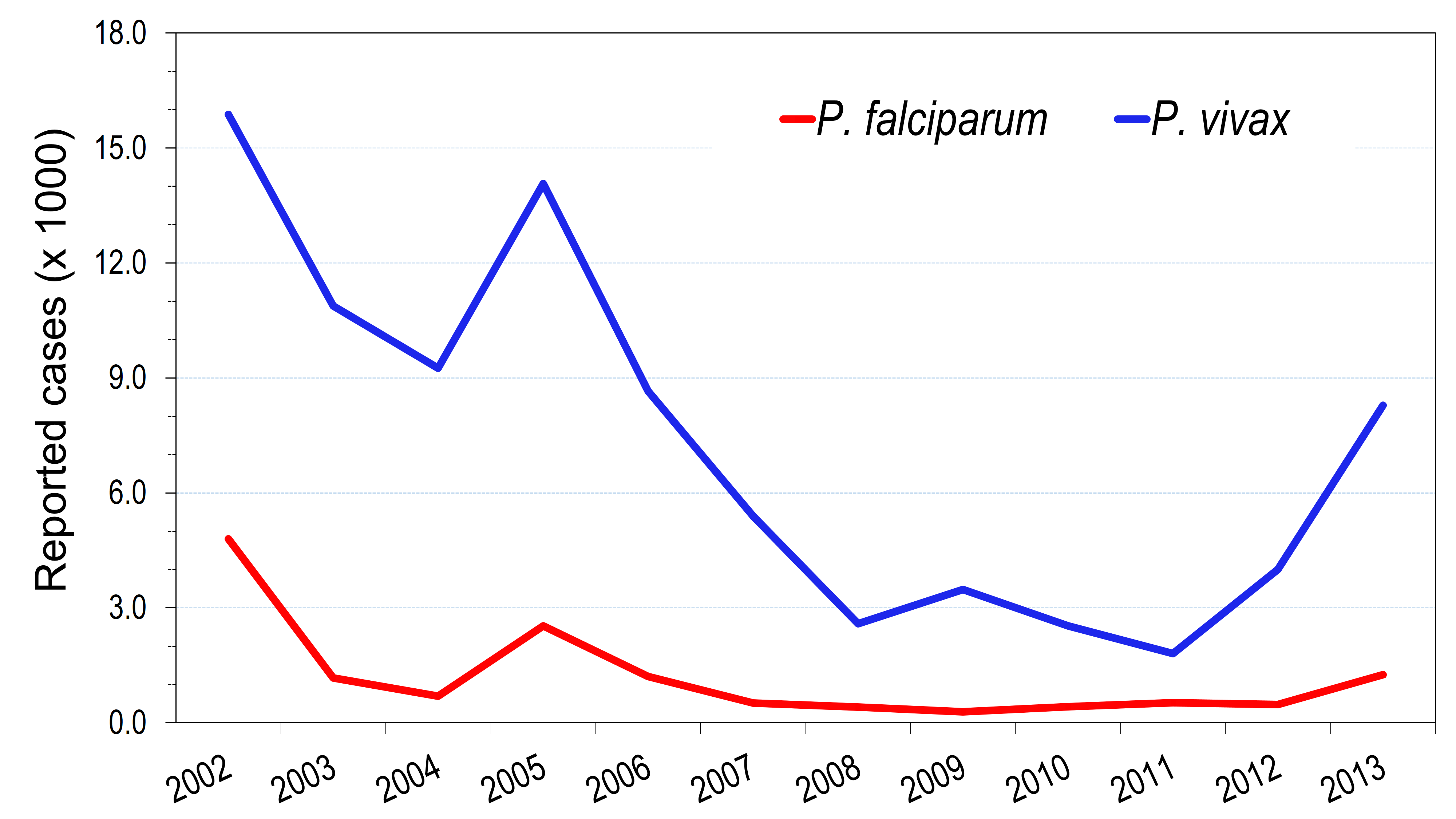

Supplement: S2 Fig — (TIF) [file pone.0137458.s002.tif]
