# Supplementary material for: Hotspots of Malaria Transmission in the Peruvian Amazon: Rapid Assessment through a Parasitological and Serological Survey
Source: PLoS One. 2015 Sep 10;10(9):e0137458. doi: 10.1371/journal.pone.0137458 (PMC4565712; doi:10.1371/journal.pone.0137458)
Supplement: S1 Table — (DOCX) [file pone.0137458.s003.docx]

**S1 Table. Survey coverage by site**

| **Site** | **Age** | **Census** | **Enrolled** | **%** |
| --- | --- | --- | --- | --- |
|  |  |  |  |  |
|  |  |  |  |  |
| Site A | < 6 m | 41 | ▁ | ▁ |
|  | 6 m - 6.9 y | 146 | 109 | 74.7 |
|  | 7-14.9 y | 149 | 42 | 28.2 |
|  | 15-30 y | 188 | 47 | 25.0 |
|  | > 30 y | 131 | 40 | 30.5 |
|  | Total | 655 | 238 | 36.3 |
|  |  |  |  |  |
| Site B | < 6 m | 31 | ▁ | ▁ |
|  | 6 m - 6.9 y | 105 | 74 | 70.5 |
|  | 7-14.9 y | 60 | 19 | 31.7 |
|  | 15-30 y | 58 | 17 | 29.3 |
|  | > 30 y | 123 | 43 | 35.0 |
|  | Total | 377 | 153 | 40.6 |
|  |  |  |  |  |
| Site C | < 6 m | 45 | ▁ | ▁ |
|  | 6 m - 6.9 y | 169 | 121 | 71.6 |
|  | 7-14.9 y | 124 | 40 | 32.3 |
|  | 15-30 y | 125 | 44 | 35.2 |
|  | > 30 y | 154 | 55 | 35.7 |
|  | Total | 617 | 260 | 42.1 |
|  |  |  |  |  |
| Total | < 6 m | 117 |  |  |
|  | 6 m - 6.9 y | 420 | 304 | 72.4 |
|  | 7-14.9 y | 333 | 101 | 30.3 |
|  | 15-30 y | 371 | 108 | 29.1 |
|  | > 30 y | 408 | 138 | 33.8 |
|  | Total | 1649 | 651 | 39.5 |
