# Supplementary material for: Hotspots of Malaria Transmission in the Peruvian Amazon: Rapid Assessment through a Parasitological and Serological Survey
Source: PLoS One. 2015 Sep 10;10(9):e0137458. doi: 10.1371/journal.pone.0137458 (PMC4565712; doi:10.1371/journal.pone.0137458)
Supplement: S2 Table — (DOCX) [file pone.0137458.s004.docx]

**S2 Table. Association between PCR results and microscopy, serology and symptoms**

|  |  | **PCR results*** | | | | | |  |  |
| --- | --- | --- | --- | --- | --- | --- | --- | --- | --- |
|  |  | **Negative** | | ***P. vivax*** | | ***P. falciparum*** | |  | **p-value** |
|  |  | n | % | n | % | n | % |  |  |
|  |  |  |  |  |  |  |  |  |  |
| **Total analyzed** | | 596 |  | 18 |  | 34 |  |  |  |
| **Microscopy** | |  |  |  |  |  |  |  |  |
|  | Negative | 594 | 99.7 | 13 | 72.2 | 24 | 70.6 |  | <0.001 |
|  | *P. vivax* | 2 | 0.3 | 5 | 27.8 | 0 | 0 |  |  |
|  | *P. falciparum* | 0 | 0 | 0 | 0 | 10 | 29.4 |  |  |
| ***P. vivax* MSP-119 serology** | |  |  |  |  |  |  |  |  |
|  | Negative | 485 | 81.4 | 10 | 55.6 | 26 | 76.5 |  | 0.043 |
|  | Positive | 111 | 18.6 | 8 | 44.4 | 8 | 23.5 |  |  |
| ***P. vivax* AMA-1 serology** | |  |  |  |  |  |  |  |  |
|  | Negative | 493 | 82.7 | 9 | 50 | 26 | 76.5 |  | 0.004 |
|  | Positive | 103 | 17.3 | 9 | 50 | 8 | 23.5 |  |  |
| **Combined *P. vivax* serology** | |  |  |  |  |  |  |  |  |
|  | Negative | 445 | 74.7 | 8 | 44.4 | 21 | 61.8 |  | 0.011 |
|  | Positive | 151 | 25.3 | 10 | 55.6 | 13 | 38.2 |  |  |
| ***P. falciparum* GLURP serology** | |  |  |  |  |  |  |  |  |
|  | Negative | 513 | 86.1 | 15 | 83.3 | 21 | 61.8 |  | 0.003 |
|  | Positive | 83 | 13.9 | 3 | 16.7 | 13 | 38.2 |  |  |
| ***P. falciparum* AMA-1 serology** | |  |  |  |  |  |  |  |  |
|  | Negative | 554 | 93 | 18 | 100 | 27 | 79.4 |  | <0.001 |
|  | Positive | 42 | 7 | 0 | 0 | 7 | 20.6 |  |  |
| **Combined *P. falciparum* serology** | |  |  |  |  |  |  |  |  |
|  | Negative | 489 | 82 | 15 | 83.3 | 18 | 52.9 |  | <0.001 |
|  | Positive | 107 | 18 | 3 | 16.7 | 16 | 47.1 |  |  |
| **Fever and/or history of fever (previous 2 days)** | | 33 | 5.6 | 3 | 16.7 | 8 | 23.5 |  | <0.001 |
|  |  |  |  |  |  |  |  |  |  |

* One mixed infection not shown (asymptomatic individual seronegative both for*P. falciparum* and *P. vivax*
